# Barriers to Diagnosis Access for Chagas Disease in Colombia

**DOI:** 10.1155/2018/4940796

**Published:** 2018-02-07

**Authors:** Mario Javier Olivera, Julián Felipe Porras Villamil, Christian Camilo Toquica Gahona, Jorge Martín Rodríguez Hernández

**Affiliations:** ^1^Grupo de Parasitología, Instituto Nacional de Salud, Bogotá, Colombia; ^2^Health Economics Program, Pontificia Universidad Javeriana, Bogotá, Colombia; ^3^Facultad de Medicina, Universidad Nacional de Colombia, Bogotá, Colombia; ^4^Dirección de Investigación en Salud Pública, Instituto Nacional de Salud, Bogotá, Colombia

## Abstract

Chagas disease is the leading cause of nonischemic cardiomyopathy in Latin America. Timely access to diagnosis and trypanocidal treatment and preventive tools for millions of infected people continues to be a challenge. The purpose of this study was to identify potential barriers for the diagnosis of Chagas disease in Colombia from the perspective of healthcare providers. Using a simultaneous mixed-methods study design, we analyzed trends in access to screening and diagnosis for Chagas disease in Colombia and assessed the national barriers to access. The main barriers to access at the national level included a limited governmental public health infrastructure for the diagnosis of Chagas disease and limited physician awareness and knowledge of the disease. Data indicate that 1.5% of total expected cases based on national prevalence estimates were reported. Few public health laboratories have the capacity to perform complementary tests for the diagnosis of Chagas disease and almost 6 months elapse between the requests of the tests and the confirmation of the disease. This study shows that infected people must overcome a number of barriers to achieve diagnosis. Reducing barriers to early diagnosis of Chagas disease is an important goal in the fight against the disease.

## 1. Introduction

Timely access to diagnosis and anti-*Trypanosoma* therapy by people living with Chagas disease has been shown to substantially reduce morbidity and mortality, as well as improve the overall quality of life [1]. American trypanosomiasis is endemic in Latin America, where it is caused by the parasite* Trypanosoma cruzi*. However, Chagas disease has become an emerging global problem due to the growing international migration and travel of Latin Americans to nonendemic countries [2]. The World Health Organization (WHO) estimates that Chagas disease affects 6-7 million people worldwide and it causes more than 7000 deaths per year [3]. Although prevalence data are limited, the most recent estimate suggests that about 437,960 people living in Colombia are infected with* T. cruzi* [3]; the vast majority of people with the disease remain undiagnosed and untreated.

The diagnosis of Chagas disease is complex due to the dynamics of parasitemia in the phases of the disease [4]. In the acute phase, the parasitemia is high, and therefore the diagnosis is performed by direct parasitological tests. Nevertheless, direct parasitological tests are not useful in the chronic phase due to the low and intermittent parasitemias [1]. Therefore, the diagnosis of Chagas disease in the chronic phase is determined by serological tests such as enzyme-linked immunosorbent assay (ELISA), indirect hemagglutination assay (IHA), indirect immunofluorescence assay (IFA), western blot, and rapid diagnostic tests such as immunochromatography [5–7]. A considerable variation in the reproducibility and reliability of the results is observed with the five methods. A single test is not sufficiently sensitive and specific to make the diagnosis. For this reason, the WHO recommends applying two or more tests that use different techniques and/or detect antibodies to different antigens [8]. The conventional serological tests commonly used are ELISA and IFA, which are time-consuming and consist of several steps, thus increasing the possibility of operational error [4].

Recently, molecular techniques such as polymerase chain reaction (PCR) have been considered as supportive diagnostic tests due to their ability to determine parasitic loads of* T. cruzi* in all clinical phases of the disease [9]. Unfortunately, its routine use in resource-poor settings is limited by the high costs and every lab needs dedicated PCR infrastructure, trained technicians, and specialized equipment, and it is not yet recommended as a screening or diagnosis test [9]. Recent technological developments have led to the proliferation of new rapid diagnostic tests based on recombinant proteins or synthetic peptides and have shown promising results for the diagnosis of* T. cruzi* infection [10]. However, these tests need to be adapted in different settings. These include differences in the characteristics of the population or the infectious agent, including the infection prevalence and genetic variation of the pathogen, as well as the test methodology [10].

In Colombia, Chagas disease testing has not been broadly promoted, as well as the etiological treatment [11, 12]; individuals are traditionally tested when they donate blood [13]. Screening in blood banks is the main source of identifying cases and the prenatal care programs as a regular practice do not recommend screening for Chagas disease in pregnant women. In addition, most healthcare providers do not routinely offer screening tests to detect* T. cruzi* infection to their patients.

This research aimed to identify potential barriers to healthcare access for Chagas disease in Colombia, defined as diagnosis, from the perspective of healthcare providers, and, additionally, to systematically review the literature on the views of healthcare professionals to the access to healthcare for Chagas disease and to identify potential barriers.

## 2. Methods

A simultaneous mixed-methods study design was conducted, involving (i) a systematic review of the literature to identify published papers describing the views on the barriers to access to healthcare for Chagas disease from the perspective of health managers and health professionals, (ii) a cross-sectional survey, and (iii) semistructured in-depth interviews, to collect data from healthcare providers and policymakers at the national level in Colombia. In addition, (iv) key documentary information was examined. The results of the four stages of data collection were formally triangulated.

### 2.1. Systematic Literature Review

#### 2.1.1. Search Strategy and Identification of Articles

This review was carried out as per PRISMA guidelines (Table 5). Extensive electronic searches were conducted for published literature in PubMed, EMBASE, SciELO, and Google Scholar. A secondary search was conducted by reviewing the reference lists of the retrieved studies. Searches were restricted to articles written in English and Spanish, but there were no publication year or status restrictions. The date of the search was 1 August 2016. See Appendix A for the full search strategy for each database.

#### 2.1.2. Study Selection

Identified articles were initially filtered with a title search by two investigators independently. Publications were included if they reported on views of health managers and health professionals on access barriers to healthcare for Chagas disease. The concept of access to healthcare was defined as the degree to which people are able to reach and obtain adequate care from the healthcare system in a timely manner [14, 15]. Review articles and research published only in abstract format were excluded. Two reviewers independently read full-text versions of eligible articles and disagreements were resolved by consensus, and in the case of persistent disagreement, input from a third reviewer was obtained. The Critical Appraisal Skills Programme checklist was used to assess the quality of the included studies [16].

#### 2.1.3. Data Extraction and Synthesis of Results

The extraction of the results from the primary studies in this review was done manually in a matrix (Access 2007, Microsoft Inc., Redmond, WA), which included data on authors, year of publication, country of origin, aims, research design, characteristics of the scenarios and participants of studies, data recognition tools, method of data analysis, and specific results presented by the researchers. Data were extracted by one reviewer and checked by two others for omissions and accuracy. The data analysis in this review was carried out following the approaches for the generation of qualitative metasynthesis suggested by* Sandelowski and Barroso* [17], through comparative analysis. These findings were classified to determine the level of congruence between the results and supporting data from primary studies as proposed by* Briggs* [18]. This classification of the findings allows identifying the degree of credibility of the researcher's interpretation in three levels: unequivocal (findings accompanied by an illustration that is beyond reasonable doubt and therefore not open to challenge), credible (findings accompanied by an illustration lacking clear association with it and therefore open to challenge), and unsupported (findings not supported by data).

### 2.2. Data Collection: Interviews

Qualitative data were collected via interviews (Appendix B). We made a qualitative explorative study with data from a purposive sample of sixteen key informants through semistructured, face-to-face interviews. The key informants, who were health policymakers, heads of departments, directors of hospitals, and primary care physicians, mainly worked at the national level, but also a few at provincial levels. These physicians had knowledge and experience in treating cases of Chagas disease. Primary care physicians included general practice, family practice, internal medicine, and obstetrics and gynecology. Health managers were selected for this study because their position usually has oversight of the budget, equipment purchasing, facility operations, and patient flow. In addition, snowball sampling, a strategy where experts help identify other information-rich cases, was used. The interviews took place in the offices of the key informants during official working hours and each interview took between 30 minutes and one hour.

### 2.3. Data Collection: Survey Research

Qualitative and quantitative data were collected via self-report. A self-administered questionnaire was created to assess the perception of these key informants about their own services in relation to barriers to access to diagnosis for Chagas disease and to evaluate the awareness and knowledge about the recommendations on the diagnosis of Chagas disease according to Colombian clinical practice guidelines (Appendix C). The questionnaire was pretested on a convenient sample of 10 health professionals (not included in the final sample). The questionnaires were distributed to the professionals during their interview. In addition to questions on sociodemographic information, the final instrument in Spanish language had 11 items. The questionnaire included quantitative and qualitative questions. The awareness was assessed using one question regarding whether the professional had previously heard about the clinical practice guideline for Chagas disease. If the professional responded positively to the question, he/she was considered aware and received 10 follow-up questions. For the five quantitative questions, the respondents answered either “yes,” “no,” or “do not know.” One point was given for each correct response and zero points for each wrong or “do not know” response on items related to knowledge. The minimum and maximum possible scores were 0 and 5, respectively. The qualitative component of the questionnaire included five open-ended questions.

### 2.4. Data Collection: Documentation

Key documentary information was examined, for example, annual reports of confirmed cases of Chagas disease between 2008 and 2015, clinical practice guidelines, and protocols and reports of the national network of public and private laboratories in Colombia, including blood banks. A range of information was obtained from websites related to the National Institute of Health and the Ministry of Health of Colombia. The information regarding the location of the different laboratories with the capacity to diagnose* T. cruzi* infection discriminated by serological tests was displayed on a map. The ESRI's ArcGIS (release 10) software was used to construct the map.

### 2.5. Data Analysis

All quantitative survey data were doubly entered into a computerized relational database (Access 2007, Microsoft Inc., Redmond, WA). Means and standard deviations (SD) were determined for quantitative variables. Data were analyzed using Fisher's exact test to compare categorical variables and an unpaired *t*-test or one-way ANOVA for continuous variables. Statistical analyses were performed using the Stata® (release 11.0) software package (Stata, College Station, TX). *P* values less than 0.05 were considered as statistically significant.

Examination was based on framework analysis and thematic analysis methods. All qualitative data obtained from in-depth interviews and policy documents were described and organized according to the Health System Reform Framework [19], which allowed identifying health system deficiencies, and the thematic analysis method also allowed identifying emerging issues. Audio tape recordings of in-depth interviews were transcribed and the data were analyzed with NVivo software (QSR International Pty Ltd., Doncaster, Australia).

### 2.6. Data Integration

Three triangulation techniques were used for the integration of research components [20]. These included methodological triangulation, with the use of more than one data collection technique (interviews, questionnaires, and documentation); data triangulation, with the use of multiple data sources (reports, physician, and health policymakers); and investigator triangulation using three researchers in the analysis. The integration of these components was arranged in the Health System Reform Framework considering three key emerging issues. Subsequently, the data sources using qualitative and quantitative methods were compared to see if they converged or diverged. The convergence of themes across the datasets was coded and assessed using triangulation matrices to display and interpret findings. “Agreement” indicates that the key finding was identified, “partial agreement” means that the finding was partially covered, and “disagreement” indicates a contradictory finding. If none of these three codes could be attributed, the label “silence” was used [20].

### 2.7. Ethical Considerations

Approvals were granted by the Technical Research Committee and Ethics Research Board at the National Health Institute in Bogotá, Colombia, Protocol CTIN-014-11, Minute 9 of December 11, 2012. Participation was voluntary and informed consent was obtained from all interviewees.

## 3. Results

### 3.1. Results of Literature Review

**Figure 1:**
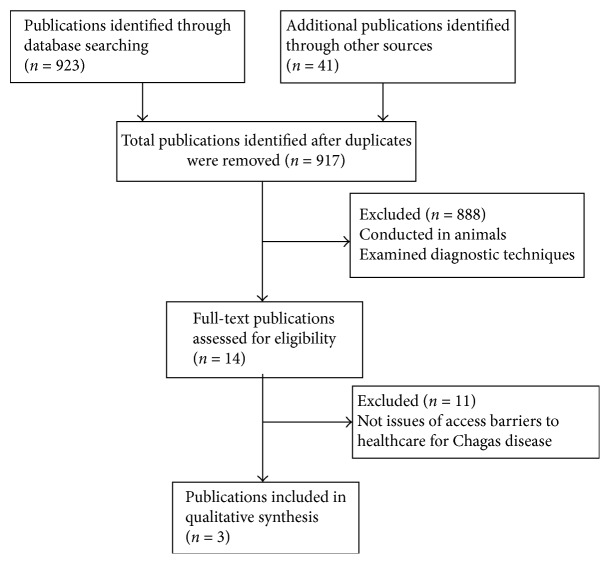
PRISMA flow diagram describing the review process and study selection.

The literature search identified 964 records. After screening titles and abstracts, 14 met inclusion criteria and were eligible. After full-text review and manual review of references, 3 publications were included; a PRISMA flow diagram of study selection is presented in Figure 1.

The revision of the articles included in this study reported that patients with Chagas disease face a variety of barriers to obtaining adequate and timely access to care for this disease, mainly barriers associated with diagnosis and treatment. Most of the views expressed about access to healthcare for a person infected with* T. cruzi* were negative. The main findings of each study are summarized in Table 1.

The most frequent barriers reported were limited diagnostic and institutionalized referral and care processes, lack of laboratories that perform confirmatory tests, lack of financing for patient-care activities, limited awareness and training among providers, lack of licensing of drugs for Chagas disease, absence of national clinical guidelines, and limited provider awareness.

### 3.2. Results of Interviews

**Figure 2:**
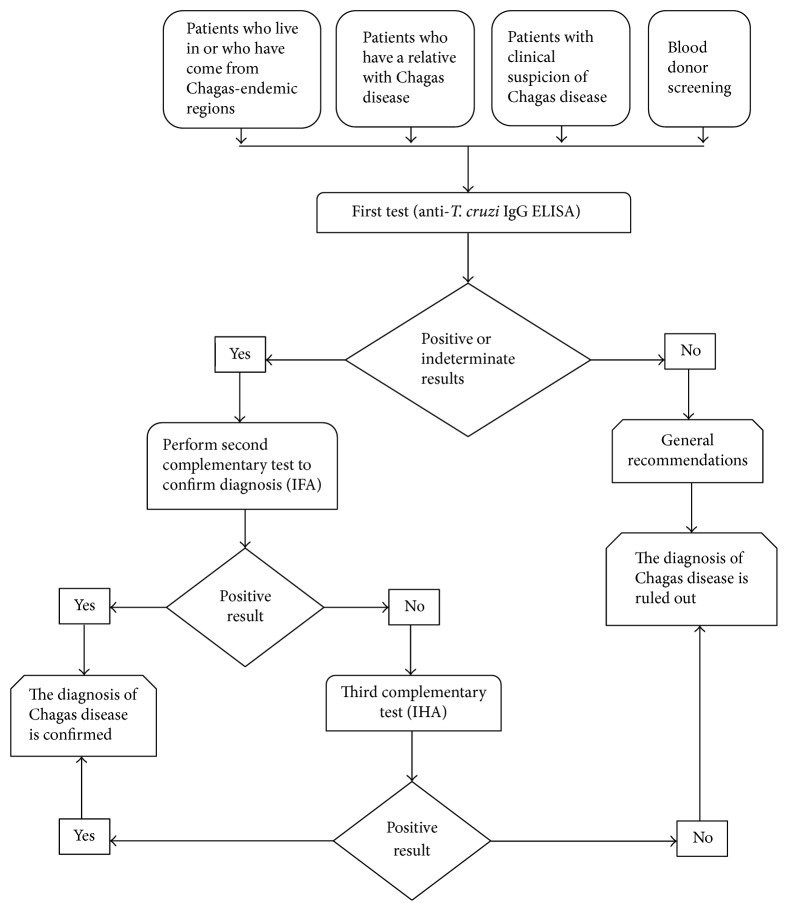
Generalized flow diagram of Chagas disease diagnosis in Colombia.

Interviews with health managers and health professionals provided information on the routes for access to the diagnosis of Chagas disease in the health system (see Figure 2) and about barriers that people face to request their diagnosis and that the organization faces in prevention and health promotion services.

Key informants unanimously declared that although the diagnosis of the disease was provided within their organization, community services and issues beyond their control also affected access to health and integration they were able to provide. Barriers to access that emerged from the key informant interviews were described and organized according to the Health System Reform Framework [19].

*Financing and Payment*. The results of the interviews suggest that financing is a fundamental barrier that limits the access to diagnosis for many patients with Chagas disease. In Colombia, tests for the diagnosis of* T. cruzi* in both the acute phase (direct methods) and the chronic phase (indirect methods) are part of the basic services package of the mandatory health plan and any person with subsidized health insurance (without ability to pay) or contributory regime (with ability to pay) can have access to them. Despite this, most patients have to bear the cost of ~$25 US dollars (USD) of the confirmatory test (IFA) due to the lack of laboratories in the primary care centers that perform this second test. In addition, patients for each medical appointment and for each test must pay a copayment (it is a percentage payment that must be made by the beneficiaries of the contributor to the contributory regime and all affiliates of the subsidized regime, ~$1 to $8 USD). Alternatively, they must pay a moderating fee (a contribution in money to be paid by all members of the contributory scheme, ~$1 to $8 USD). In addition, patients must bear transportation costs to primary care centers (~$3 USD) and the displacement towards the laboratories (~$3 to $25 USD) that are usually found in cities. On the other hand, coverage rates for health affiliation are less than 100%, especially in rural areas. People without health affiliation experience greater barriers to care, delay seeking care, and have greater unmet needs.

*Organization*. Interviewees indicated that the greatest organizational challenges were the absence of diagnostic tests in hospitals in primary care, lack of validation of rapid tests, high heterogeneity in test reliability, few centers with the capacity to perform confirmatory tests, and the nonintegration of diagnosis and treatment of Chagas disease in primary healthcare centers, which are barriers affecting access to diagnosis. In Colombia, there is a national network of laboratories composed of 4 national reference laboratories, 32 departmental public health laboratories, and 1 district public health laboratory. These laboratories are public institutions responsible for carrying out diagnostic activities, technical and educational activities, and quality control and providing support, reference, and counter-reference for public health surveillance and disease control. However, laboratories that can perform confirmatory testing are very few. On the other hand, blood banks have an obligation to screen all blood units for* T. cruzi* and confirm all seroreactive units. Nevertheless, there is no follow-up of positive cases. There is a guideline for the diagnosis and management of Chagas disease that has important limitations in the applicability, mainly because most patients with this disease are located in rural areas where there are organizational barriers that prevent the implementation of recommendations. In addition, the guideline has not been updated with the available evidence against* T. cruzi *infection, which prevents its use as a reliable tool for clinical decision-making.

*Regulation*. Respondents indicated that in order to perform diagnostic tests for Chagas disease patients must face some administrative barriers. The first barrier that patients face is to get a medical appointment for which they must call by phone. In this first step, the interviewees indicate that telephone lines are often busy or patients are redirected. Subsequently, the tests must be authorized by the health provider and the patient is directed to the laboratory. Again, the patient should contact the laboratory for the tests. These are the steps that a person belonging to the subsidized or contributory health regime must follow. In general, the interviewees report that the time elapsed since the physician requests the tests until confirming the diagnosis is 6 months.

*Behavior*. There is a general agreement among the interviewees that physicians in Colombia are aware of the existence of the disease and have a good knowledge of Chagas disease. However, they believe that physicians have weaknesses in diagnosis. They are unaware of the diagnostic tests that are available in the country and have difficulty interpreting the results. Another problem is that there is an increasing tendency to specialize the disease (cardiologists and infectious disease specialists, among others) and patients are not treated in the primary care centers. Resistance persists by physicians to consider this disease as a possible diagnosis in the population at risk. It is necessary to keep updating these professionals by emphasizing the diagnostic interpretation. Barriers to access to diagnosis for Chagas disease are summarized in Table 2.

### 3.3. Results of Survey Research

In total, 16 key informants were included. All questionnaires were completed and returned. The mean age of the respondents surveyed was 48.4 and the average number of years as an administrator or physician was 11.4. The majority of the respondents were male (12, 66.7%). All respondents were familiar with the existence of a guideline for the diagnosis and management of Chagas disease; the score obtained was associated with age and experience of the respondent; see Table 3.

The group (health professionals and health managers) evaluated that 100% of the population that they cover have access to the diagnosis for* T. cruzi *in the acute phase of the disease and only 50% of this population have access to the diagnosis in the chronic phase. All believe that the diagnosis for this disease should be available in primary care centers and all agree on the importance of guidelines to facilitate decision-making.

The health managers refer that their institutions have an e-mail, telephone, and access to the Internet and this allows physicians to review the diagnostic and management guideline. The institutions do not have the infrastructure or economic resources to acquire the necessary equipment to confirm the diagnosis. They report that they have heard of other diagnostic methods such as rapid tests, but these are not recommended by the guidelines. In addition, they believe that other strategies should be used to explain the content of the guidelines because doctors “are saturated with clinical practice guidelines.” They perceive resistance from physicians to consider this diagnosis and they refer discomfort to research projects that screen for Chagas disease and do not disclose the results to the participants.

The health professionals feel that the diagnosis of Chagas disease is difficult because of the lack of specific signs or symptoms, especially in the adult population that generally has other comorbidities, and also the report of the results is not standardized and changes according to the laboratory, making the interpretation of the results difficult, especially “indeterminate results.” With regard to guidelines, they consider that these are rarely applicable to clinical practice and that the recommendations are not explicit and contain many tests that are not available in their region. They feel uneasy with research projects that perform specialized tests that they cannot interpret and that are not considered as diagnostic tests.

### 3.4. Results of Review of Documents from Colombia

In Colombia, it is estimated that there are 4,813,543 people at high risk for getting Chagas disease and 437,960 cases infected with* T. cruzi* [3]. A total of 65,200 tests were performed for screening of Chagas disease between 2008 and 2015, representing 1.35% coverage of screening of the population at risk for this period. Of these, 6,722 (10.3%) cases were confirmed with infection by* T. cruzi* according to the Weekly Epidemiological Bulletin of the National Institute of Health [24]. This is equivalent to 1.5% of the total cases estimated by the WHO. On the other hand, the data reported by the National Blood-Banks Network showed that 5,134,191 blood units were screened for* T. cruzi,* representing 100% coverage of screening in donors for the same period. 0.41% and 0.38% of blood units were reactive for* T. cruzi *during 2014 and 2015, respectively [25].

In addition, a predominance of cases with chronic Chagas disease was observed in the main cities of Colombia. However, the data from the blood banks come from a selected population and these do not represent the distribution of the disease in the country. Unfortunately, there is no information on cases confirmed by blood banks and their referral to primary care centers.

Regarding the clinical practice guideline [26], it was observed that it was elaborated in 2009, it has not been updated, and it has methodological limitations due to the lack of standardized processes in its development, which resulted in a guideline with an overall quality of moderate to low [27, 28].

**Figure 3:**
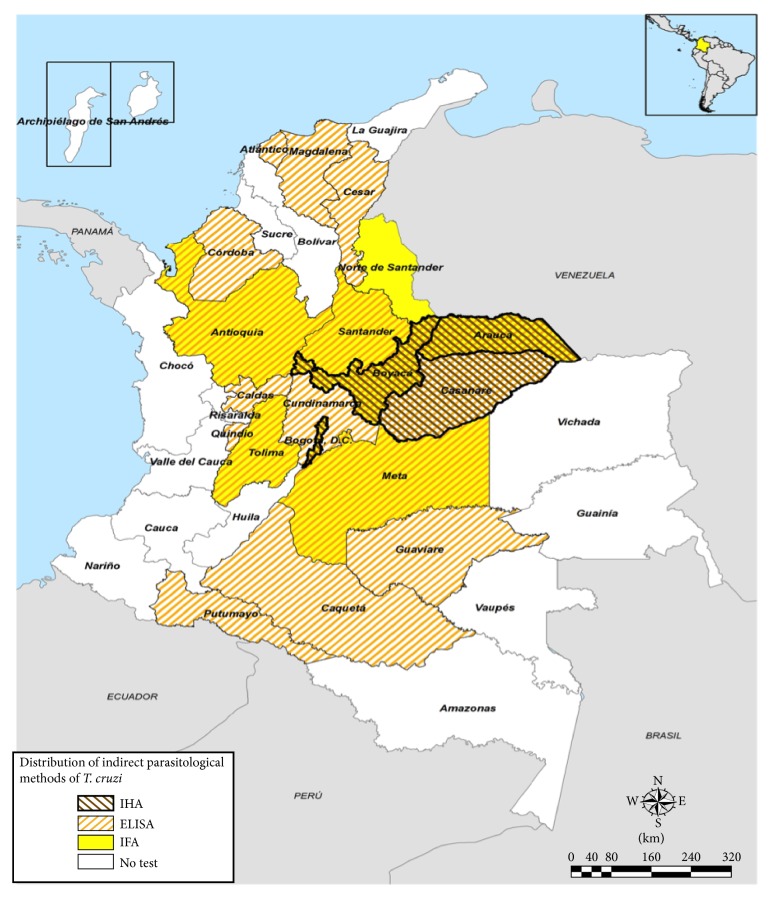
Distribution of laboratories according to department with the capacity to perform indirect parasitological tests (ELISA, IFA, and IHA) for diagnosis of Chagas disease in Colombia.

With regard to the diagnosis of Chagas disease in Colombia, all 33 public health laboratories have the capacity to carry out direct parasitological methods (thick and thin peripheral blood drop). However, of the 33 public health laboratories, only twenty have the structural and technological capacity to perform at least one indirect parasitological test (ELISA, IFA, or IHA), five laboratories perform 2 diagnostic techniques, and three laboratories perform 3 indirect parasitological tests. Eighteen public health laboratories perform ELISA, seven laboratories perform IFA, and three laboratories perform IHA. Distribution of indirect parasitological methods for diagnosis of Chagas disease in Colombia is shown in Figure 3.

### 3.5. Results of Integration Data

A total of 14 key findings were identified and the level of agreement among the sources of information is shown in Table 4. The findings showed an almost perfect agreement between the views of health professionals and health managers (13/14, 93%). There was no disagreement on any findings among the sources of information, but the literature review and document review had a “silence” coding. All but two of the findings identified in the interviews and questionnaires were further identified in the systematic review. Quantitative and qualitative data about health professionals and health managers showed that these professionals converge in most of their perceptions.

## 4. Discussion

This study provides evidence to suggest a wide gap in access to the diagnosis of Chagas disease in Colombia. To measure the gap in access to diagnosis, this research shows that from 2008 to 2015 only a small percentage (1.5%) of the estimated cases in Colombia were identified through the public health surveillance system [20]. There is no information regarding the number of cases identified in blood banks, only data on the percentage of seroreactivity to* T. cruzi* in blood units. However, it is known that the referral and counter-referral system between blood banks and primary care centers for public health surveillance is deficient, which limits the follow-up of these cases. A similar situation occurs in the United States where less than 1% of the estimated cases were identified among blood donors, the only source of national case data [17].

This research also clearly shows the barriers to access to health services faced by patients with Chagas disease in different regions of the world, in particular, the barriers related to access to diagnosis and treatment of the disease. The interviewees confirmed that almost 6 months elapse between the requests of the tests and the confirmation of the diagnosis of the disease. The health system barriers that explain the gaps in access to care for patients with Chagas disease can be divided into three main barriers: (1) limited diagnosis of Chagas disease, (2) funding, and (3) limited physician awareness and knowledge of the disease.

The first is related to the limitations of access to diagnosis, a barrier identified in several regions of the world like the United States [21], Mexico [23], Italy [29], and Switzerland [30]. This barrier is closely related to the degree of knowledge about Chagas disease [31]. If the knowledge of the physicians is limited, they will not consider this disease as a potential diagnosis. In addition, the lack of clear recommendations for screening, the requirement of two tests to confirm the diagnosis, the lack of laboratories with the capacity to perform the two tests in primary care hospitals, and the few centers to perform confirmatory tests are all potential factors that limit access to diagnosis. The present research shows that in Colombia there are few public health laboratories with the capacity to perform the two complementary tests for the diagnosis of the disease.

In order to reduce this barrier, countries have marketed rapid tests. However, in Mexico and the United States, great heterogeneity has been observed in the results [21, 23]. In contrast, Colombia has not validated the use of these tests for routine clinical practice. In addition, some countries have established screening in blood banks as mandatory; in countries such as Colombia and the United States, the screening rate is 100% [13, 21]. Other countries such as Mexico, where Chagas disease is endemic, have not been able to complete this percentage [23]. Moreover, other barriers coexist such as immigration status, high costs to access to the health system, and language, which perpetuate the gap to access to the diagnosis [21].

The second limitation is funding. Financial barriers, not surprisingly, played a central role as demonstrated by several studies in the United States [21] and Mexico [23], as well as studies in other European countries, such as Switzerland [32] and Italy [29]. Interviews with health managers and health professionals indicate that although the diagnosis of Chagas disease is covered by the health benefits plan, to carry out these tests, health insurance companies must previously authorize the service, which generates dissatisfaction to users by the queues, time consumption, distances to the authorization sites, denials, and systematic delays by health insurers. These results are in agreement with those reported by Hernández et al. [33], who characterized the main administrative barriers faced by the Colombian population when they attempt to gain access to health services. They found the barriers derived from the authorizations, lack of opportunity for specialized medical appointments, surgical procedures, and drug delivery. These researchers also reported delays in assigning appointments; they found that telephone lines were busy or users were referred repeatedly for appointment assignment. To this worrying situation, the bureaucratic and administrative barriers existing in authorizations must be added.

The third limitation is the lack of awareness and knowledge among physicians and patients about the disease. According to the interviewees, the lack of awareness of the disease represents one of the great barriers that limit access to diagnosis. If the physicians are not aware of the existence of the disease, they will not consider it as a potential diagnosis in the population at risk and will not order the needed diagnostic tests. This finding is similar to others reported in the United States [31], indicating that the lack of training of physicians is a possible risk factor for late diagnosis of the disease. In European countries, being nonendemic regions, this aspect becomes a determining factor for a timely diagnosis [29, 32]. In Mexico, although information is being expanded and circulated through various channels, in most cases, it does not promote concrete actions [23]. In Colombia, the government has carried out educational campaigns to strengthen medical skills and raise public awareness about the disease, but it has not evaluated the impact of these campaigns. In addition, this study shows the difficulties that physicians have with respect to the interpretation of test results and the tendency to specialize the disease.

Moreover, key informants perceived the inadequate implementation of clinical practice guidelines for Chagas disease. Most of the respondents mentioned that the guidelines are available on the website of the Ministry of Health of Colombia, but they lacked detailed knowledge of them probably because the guidelines as well as control programs were not widely disseminated. The respondents agreed that education about Chagas disease and control programs should be integrated in the training curricula, especially in medical schools and primary schools in endemic regions.

This study has some limitations; first, the review of the literature only chose articles published in English or Spanish. Secondly, the data on the prevalence of Chagas disease are limited worldwide. Third, there is little information on control programs and education of Chagas disease. Fourthly, the results for Colombia were based on opinions and experiences of key informants. However, high levels of agreement between the different key informants suggest reliability of the findings. The views and opinions of key informants may not be representative of all experts of Chagas disease in Colombia.

In conclusion, this study shows that the barriers to access to healthcare for patients with Chagas disease are a global problem; vast majority of people were diagnosed late due to these gaps in access to care. The limited awareness and knowledge of physicians about the disease and the limited number of laboratories with the capacity to perform confirmatory tests are the main barriers to accessing the diagnosis of Chagas disease. Rapid tests are techniques that could help reduce the diagnostic gap. However, having rapid tests is not enough, as many barriers may prevent their successful implementation, for which we recommend qualitative research on diagnostic practices that may help identify potential barriers. In Colombia, the results of this study show that infected people must overcome a number of barriers to achieve the diagnosis. Reducing barriers to early diagnosis of Chagas disease is an important goal in the fight against the disease. However, the current barriers must be addressed in order to provide widespread access to early diagnosis.

## Figures and Tables

**Table 1:** Main characteristics and study design of the articles included in this review.

Authors (Ref)	Year of publication	Country	Study design	Key findings	CAPS
Manne-Goehler et al. [21]	2015	United States of America	Mixed methods	(1) An inability to place orders for Chagas disease diagnostic tests in institutional laboratory ordering systems, (2) heterogeneity in available diagnostic tests, (3) a limited capacity to conduct definitive confirmatory diagnostic testing, (4) poor follow-up of positive blood donors, (5) diagnostic and institutionalized referral and care processes, (6) lack of financing for patient-care activities, and (7) limited awareness and training among providers	Mixed methods

Manne-Goehler et al. [22]	2014	Mexico	Mixed methods	(1) Lack of market authorization for benznidazole, (2) long waiting times for medicine importation, and (3) limited awareness of the disease among both physicians and patients	Mixed methods

Manne et al. [23]	2013	Mexico	Mixed methods	(1) Exclusion of antitrypanosomal medicines from the national formulary, (2) historical exclusion of Chagas disease from the social insurance package, (3) absence of national clinical guidelines, (4) limited provider awareness, (5) no national clinical guidelines for Chagas disease treatment, (6) global supply chain problems: long waiting times, and (7) insufficient training and education of providers about Chagas disease and its diagnosis and treatment	Mixed methods

Ref: reference; CAPS: Critical Appraisal Skills Programme checklist.

**Table 2:** Summary of barriers to diagnosis access for Chagas disease in Colombia.

Barriers to access diagnosis
(i) Lack of diagnostic tests in hospitals in primary care
(ii) Few centers that perform confirmatory tests
(iii) Lack of awareness and knowledge about the disease among physicians
(iv) Lack of validation of rapid tests
(v) High heterogeneity in test reliability
(vi) Nonintegration of diagnosis and treatment of Chagas disease
(vii) Prior authorizations by health insurance institutions
(viii) Difficulty for physicians to interpret test results
(ix) The recruitment of medical and nursing staff
(x) Lack of insurance reimbursement for services rendered
(xi) Budget cuts
(xii) The lack of more diverse staff to serve language minority communities
(xiii) The multihiring services in different locations
(xiv) Space limitations for medical equipment
(xv) Cultural barriers
(xvi) Internal armed conflict

**Table 3:** Distribution and comparison of knowledge scores about access to care for Chagas disease according to demographic characteristics of the respondents (*n* = 16).

Characteristics	Knowledge score	
	Mean ± SD	*P* value
*Respondent*		
Administrator	3.55 ± 0.50	0.629
Physician	3.67 ± 0.53	
*Age (years)*		
40–45	4.00 ± 0	0.043
46–50	3.54 ± 0.52	
51–55	3.50 ± 0.57	
*Working region *		
Endemic	3.87 ± 0.35	0.057
Nonendemic	3.40 ± 0.52	
*Experience (years)*		
9-10	4.00 ± 0	0.028
11-12	3.50 ± 0.53	
13-14	3.40 ± 0.55	

**Table 4:** Integration of the main findings across the constitutive studies.

Key finding	Health professionals	Health managers	Literature review	Documentation
Diagnosis not considered by physicians	Agreement	Agreement	Agreement	Silence
Unclear screening recommendations	Agreement	Agreement	Agreement	Agreement
Lack of diagnostic tests in hospitals in primary care	Agreement	Agreement	Agreement	Agreement
Few laboratories that perform confirmatory tests	Agreement	Agreement	Agreement	Agreement
Lack of awareness and knowledge among physicians and patients	Agreement	Agreement	Agreement	Silence
Positive blood donors not referred to the primary care centers	Agreement	Agreement	Agreement	Partial agreement
Lack of validation of rapid tests	Agreement	Agreement	Agreement	Agreement
High heterogeneity in test reliability	Agreement	Agreement	Agreement	Partial agreement
Prior authorizations by health insurance institutions	Agreement	Agreement	Agreement	Agreement
Difficulty for physicians to interpret test results	Agreement	Agreement	Silence	Silence
The lack of more diverse staff to serve language minority communities	Partial agreement	Agreement	Agreement	Silence
The multihiring services in different locations	Agreement	Agreement	Agreement	Silence
Cultural barriers	Agreement	Agreement	Agreement	Silence
Internal armed conflict	Agreement	Agreement	Silence	Silence

**Table 5:** PRISMA Checklist.

Section/topic	#	Checklist item	Reported on page #
*Title *			
Title	1	Identify the report as a systematic review, meta-analysis, or both.	

*Abstract *			
Structured summary	2	Provide a structured summary including, as applicable, background; objectives; data sources; study eligibility criteria, participants, and interventions; study appraisal and synthesis methods; results; limitations; conclusions and implications of key findings; systematic review registration number.	

*Introduction *			
Rationale	3	Describe the rationale for the review in the context of what is already known.	
Objectives	4	Provide an explicit statement of questions being addressed with reference to participants, interventions, comparisons, outcomes, and study design (PICOS).	

*Methods *			
Protocol and registration	5	Indicate if a review protocol exists and if and where it can be accessed (e.g., web address) and, if available, provide registration information including registration number.	
Eligibility criteria	6	Specify study characteristics (e.g., PICOS, length of follow-up) and report characteristics (e.g., years considered, language, and publication status) used as criteria for eligibility, giving rationale.	
Information sources	7	Describe all information sources (e.g., databases with dates of coverage, contact with study authors to identify additional studies) in the search and date last searched.	
Search	8	Present a full electronic search strategy for at least one database, including any limits used, such that it could be repeated.	
Study selection	9	State the process of selecting studies (i.e., screening, eligibility, included in systematic review, and, if applicable, included in the meta-analysis).	
Data collection process	10	Describe method of data extraction from reports (e.g., piloted forms, independently, and in duplicate) and any processes for obtaining and confirming data from investigators.	
Data items	11	List and define all variables for which data were sought (e.g., PICOS, funding sources) and any assumptions and simplifications made.	
Risk of bias in individual studies	12	Describe methods used for assessing risk of bias of individual studies (including specification of whether this was done at the study or outcome level) and how this information is to be used in any data synthesis.	
Summary measures	13	State the principal summary measures (e.g., risk ratio, difference in means).	
Synthesis of results	14	Describe the methods of handling data and combining results of studies, if done, including measures of consistency (e.g., *I*^2^) for each meta-analysis.	

## Appendix

### A. Full Search Strategy

#### A.1. Search Performed on PubMed

1. Chagas disease: 11555
2. *Trypanosoma cruzi*: 10353
3. Healthcare disparities: 10891
4. Health services accessibility: 94450
5. Quality of healthcare: 5777583
6. Healthcare quality, access, and evaluation: 6269340
7. Chagas disease/diagnosis: 1840
8. Delivery of healthcare: 913929
9. Health services accessibility/organization and administration: 16540
10. Quality of healthcare/organization and administration: 157674
11. Healthcare quality, access, and evaluation/organization and administration: 294747
12. Delivery of healthcare/organization and administration: 162632
13. Qualitative research: 31691
14. (1) or (2) and (4): 15
15. (1) or (2) and (5): 2766
16. (1) or (2) and (6): 2807
17. (1) or (2) and (8): 96
18. (1) or (2) and (9): 4
19. (1) or (2) and (10): 23
20. (1) or (2) and (11): 35
21. (1) or (2) and (12): 11
22. (1) or (2) and (13): 3
23. (1) or (2) and (4) or (5) or (6) or (8): 6349697
24. (1) or (2) and (4) or (5) or (6) or (8) and (13): 27929
25. (1) or (2) and (9) or (10) or (11) or (12): 325121
26. (1) or (2) and (9) or (10) or (11) or (12) and (13): 3366
27. (1) or (2) and (4) or (5) or (6) or (8) and (9) or (10) or (11) or (12): 297991
28. (1) or (2) and (4) or (5) or (6) or (8) and (9) or (10) or (11) or (12) and (13): 968
29. Limit (28) to English and Spanish languages and humans: 850

#### A.2. Search Performed on EMBASE

1. Chagas disease: 22906
2. *Trypanosoma cruzi*: 13804
3. Healthcare disparities: 18859
4. Health services accessibility: 5453
5. Quality of healthcare: 446069
6. Healthcare quality, access, and evaluation: 5778
7. Chagas disease/diagnosis: 6754
8. Delivery of healthcare: 29166
9. Health services accessibility/organization and administration: 124
10. Quality of healthcare/organization and administration: 10781
11. Healthcare quality, access, and evaluation/organization and administration: 11003
12. Delivery of healthcare/organization and administration: 5709
13. Qualitative research: 115133
14. (1) or (2) and (4): 5
15. (1) or (2) and (5): 40
16. (1) or (2) and (6): 171
17. (1) or (2) and (8): 17
18. (1) or (2) and (9): 5
19. (1) or (2) and (10): 5
20. (1) or (2) and (11): 5
21. (1) or (2) and (12): 5
22. (1) or (2) and (13): 5
23. (1) or (2) and (4) or (5) or (6) or (8): 612803
24. (1) or (2) and (4) or (5) or (6) or (8) and (13): 19359
25. (1) or (2) and (9) or (10) or (11) or (12): 14716
26. (1) or (2) and (9) or (10) or (11) or (12) and (13): 416
27. (1) or (2) and (4) or (5) or (6) or (8) and (9) or (10) or (11) or (12): 36356
28. (1) or (2) and (4) or (5) or (6) or (8) and (9) or (10) or (11) or (12) and (13): 634
29. Limit (28) to English and Spanish languages and humans: 543

#### A.3. Search Performed on SciELO

Chagas OR Healthcare OR Sistemas OR Disparities OR Disparidad OR Access OR Acceso OR Barriers OR Barrera.

#### A.4. Search Performed on Google Scholar

Chagas disease AND Access.

### B. Guide for Semistructured Interview with the Sample of Key Informants

*Sample Interview Plan*

*Introduction of Interviewer. *Hello, my name is ___ (Interviewer's name) ____, and I have been asked to* identify the barriers of access for the diagnosis of Chagas disease in Colombia and that will be used in the construction of possible solutions to improve access to diagnosis*. During the interview, I would like to discuss the following topics: financing and payment, organization, regulation and behavior.

You agree to participate in this project, whose conditions are as follows:

- The project is aimed to identify the barriers to access for the diagnosis of Chagas disease in Colombia. For this purpose, semi-structured interviews will be conducted with key informants.
- Interviews will last about 30 minutes and questions will address issues of access to diagnosis.
- The interview you give and the information it contains will be used only for the purposes defined by the project.
- At any time, you may refuse to answer certain questions, discuss certain topics, or even put an end to the interview.
- To facilitate the work of the interviewer, the interview will be recorded. However, recoding will be destroyed as soon as it has been transcribed.
- All interview data will be handled so as to protect your confidentiality. Therefore, no names will be mentioned and the information will be coded.
- All data will be destroyed at the end of the project.
- * Do you agree to participate?___* (Interviewer response) _____

*Script*

- What are the routes to access the diagnosis of Chagas disease in Colombia?
- How is the process in a health care institution for a patient to access the diagnosis of chronic Chagas disease in Colombia?
- What is the average time it takes for a patient to access the diagnosis of Chagas disease in the Colombian health system?
- What are the barriers faced by patients to access the diagnosis of Chagas disease in Colombia in terms of financing and payment?
- What are the barriers faced by patients to access the diagnosis of Chagas disease in Colombia in terms of institutional organization?
- What are the barriers faced by patients to access the diagnosis of Chagas disease in Colombia in terms of regulation?
- What are the barriers faced by patients to access the diagnosis of Chagas disease in Colombia in terms of behaviors?

### C. Questionnaire on Access Barriers for the Diagnosis of Chagas Disease

This questionnaire is part of a study whose main purpose is to provide relevant information to contribute to identify the barriers to access for the diagnosis of Chagas disease in Colombia and that can be used in the construction of possible solutions to improve access to diagnosis. We expect the survey to last approximately 15 minutes. All information collected will be kept confidential. Are you willing to participate in this study?

  Yes □
  No □

*(I) Identification Data*. The first questions are for general information.

(1.1) Date of the interview: _________________ (day/month/year)
(1.2) Initials of the respondent: _________________
(1.3) Sex
    Male □
    Female □
(1.4) Age: _______________
(1.5) Studies carried out
    Undergraduate: ____________
    Postgraduate: _____________________
    ___________________________________________________
(1.6) Institution where you work: _________________________
(1.7) Place: ______________________ (municipality/department)
(1.8) Years of work experience: _____________________
(1.9) Start time of interview:_________________

*(II) Knowledge about the Diagnosis of Chagas Disease*

(2.1) Do you know the guide to clinical care for Chagas disease in Colombia?
    Yes □
    No □* Go to (3.1)*
(2.2) Is the diagnosis of Chagas disease in the acute phase carried out using direct parasitological methods?
    Yes □
    No □
    Do not know □
(2.3) Is the diagnosis of Chagas disease in the chronic phase carried out using serological methods?
    Yes □
    No □
    Do not know □
(2.4) To confirm the diagnosis of Chagas disease in the chronic phase do two tests of different principles be required?
    Yes □
    No □
    Do not know □
(2.5) Is the Polymerase Chain Reaction (PCR) technique recommended in the diagnostic algorithm for chronic phase Chagas disease in Colombia?
    Yes □
    No □
    Do not know □
(2.6) Are rapid tests recommended in the diagnostic algorithm for chronic Chagas disease in Colombia?
    Yes □
    No □
    Do not know □

*(III) Perception of Access to the Diagnosis of Chagas Disease*

(3.1) What are the routes for access to the diagnosis of Chagas disease in Colombia?
(3.2) What are the recommended tests for the diagnosis of Chagas' disease (acute and chronic) in Colombia and which are available in the Health System?
(3.3) What barriers do you consider limit access to the diagnosis of Chagas' disease in Colombia?
(3.4) What do you think about the coverage of the diagnosis of Chagas disease in your institution?
(3.5) What is your perception about the knowledge about Chagas disease that the directors and/or doctors working in your institution have?
(3.6) End time of interview:_________________

*Thanks again for participating in this project*.
